# Nasopharyngeal Angiofibroma in Adults: Age-Stratified Stage Distribution, Clinical Outcomes, and Exploratory Tumor–Normal Genomics

**DOI:** 10.3390/cancers18193180

**Published:** 2026-10-02

**Authors:** Haoyuan Xu, Huankang Zhang, Wanpeng Li, Shixing Zheng, Xiaole Song, Jingjing Wang, Quan Liu, Dehui Wang, Zhuofu Liu

**Affiliations:** Department of Otolaryngology-Head and Neck Surgery, Eye & ENT Hospital of Fudan University, 83 Fenyang Road, Shanghai 200031, China

**Keywords:** nasopharyngeal angiofibroma, adult, Radkowski stage, recurrence, clinical phenotype, whole-genome sequencing

## Abstract

Background/Objectives: Nasopharyngeal angiofibroma (NA) is generally benign, but recurrent disease and rare malignant transformation remain clinically relevant beyond adolescence. We examined adult clinical presentations, records of malignant transformation, and age-related differences in stage distribution. Methods: We retrospectively reviewed 289 surgically treated patients from January 2006 through June 2026. Radkowski stage distributions were compared between patients aged > 30 and ≤30 years using exact tests. Detailed clinical records were reviewed for 16 adults, and whole-genome sequencing (WGS) annotation summaries were examined for one tumor–blood pair. Results: Twenty-five patients (8.7%) were older than 30 years; stage was available for 287 patients. In the detailed adult subgroup, a prior pathology-slide record described focal malignant transformation with a Ki-67 index of approximately 50% in one recurrent tumor. Two other patients had database transformation entries without accompanying pathological documentation. Four recurrences and three deaths were recorded, all in stage IIIB disease. Three men had colorectal or rectal cancer histories. Low-, intermediate-, and advanced-stage tumors accounted for 36.0%, 36.0%, and 28.0% of adult cases, compared with 17.6%, 57.3%, and 25.2% of younger cases (*p* = 0.0493). The genomic annotations showed a C>A-predominant substitution spectrum. Conclusions: Adult NA included recurrent stage IIIB disease and a documented record of focal malignant transformation. Stage distributions differed between age groups. These clinical observations support pathological reassessment of recurrent tumors. The single-case genomic findings remain descriptive annotations.

## Simple Summary

Nasopharyngeal angiofibroma is a benign, locally invasive tumor that can be difficult to treat at an advanced stage. Malignant transformation has rarely been reported. We reviewed 289 surgically treated patients, including 25 older than 30 years. Among 16 adults with detailed records, a prior pathology-slide record described focal malignant transformation in one recurrent tumor, with a Ki-67 index of approximately 50%. Two other patients had database entries for transformation without accompanying pathological documentation. Four recurrences and three deaths were recorded, all in patients with stage IIIB disease. Adults had a higher proportion of low-stage tumors and a lower proportion of intermediate-stage tumors than younger patients. The genomic analysis summarized variant annotations from one adult. These findings highlight the clinical course of recurrent adult disease and the importance of pathological reassessment.

## 1. Introduction

Nasopharyngeal angiofibroma (NA), historically termed juvenile nasopharyngeal angiofibroma (JNA), is a rare fibrovascular tumor that occurs predominantly in adolescent males. Although histologically benign, it can extend from the sphenopalatine region into the pterygopalatine and infratemporal fossae, orbit, cavernous sinus, and intracranial compartment. The Radkowski system describes this anatomical extent [1]. Local invasion or recurrence does not, by itself, establish malignancy. Nevertheless, malignant transformation has been reported, including in radiation-naive disease; this distinction is particularly relevant when an adult presents with a changing or repeatedly recurrent tumor [2].

Most epidemiological and clinical series concern adolescents and young adults [3,4], although early childhood presentations have also been reviewed [5]. Evidence for NA diagnosed after 30 years of age comes mainly from case reports and small reviews [6,7]. Reports of delayed recurrence also document persistence beyond adolescence [8]. In adults, the differential diagnosis includes other vascular, epithelial, neuroendocrine, and mesenchymal skull-base tumors. Age at treatment and age at disease onset are distinct clinical variables.

Molecular studies of NA have reported diverse signaling and genomic alterations, but most evidence comes from adolescent or young-adult cohorts [3,9]. Whole-exome sequencing in younger patients has also identified recurrent variants, including variants on chromosome Y [10]. Molecular data from older patients remain scarce. Activating CTNNB1 mutations were reported in 12 of 16 sporadic tumors by Abraham et al. [11]. The reported association between NA and familial adenomatous polyposis (FAP) also makes personal and family colorectal cancer histories clinically relevant [12].

We compared Radkowski stage distributions across age groups in a single institutional NA cohort, characterized a nested adult subgroup with detailed clinical records, and examined tumor–blood WGS annotations from one adult. The clinical review focused on recurrent disease and records of malignant transformation. Pathological findings and database entries were reported separately.

## 2. Materials and Methods

### 2.1. Study Design and Cohort Construction

This retrospective study was conducted at the Eye & ENT Hospital of Fudan University, a tertiary rhinology and skull-base referral center. A single institutional clinical database was queried for surgically treated patients recorded from January 2006 through June 2026. The query returned 298 JNA-coded surgical rows and 18 additional records with diagnoses other than confirmed NA. The 18 nonconfirmed records were excluded after diagnostic review and were not analyzed further. Eligible JNA records required surgical treatment at our institution, a histopathological diagnosis of NA, and an available age at operation. Three rows without a usable patient identifier were excluded. Records were deduplicated by hospital number, with patient name used only when a hospital number was unavailable. Six repeat-procedure rows were consolidated, yielding 289 unique patients for patient-level analysis.

Patients were classified by age at the index operation as >30 years or ≤30 years. Detailed clinical data were available for 16 patients aged > 30 years. This subgroup was selected according to record completeness and analyzed descriptively. The molecular analysis included paired tumor and peripheral-blood WGS summaries from one 60-year-old man. Age was also analyzed continuously and using alternative thresholds.

### 2.2. Clinical Variables and Outcomes

Age and Radkowski stage (IA–IIIB) were extracted from the patient-level cohort [1]. Stages were grouped as low (IA–IB), intermediate (IIA–IIC), and advanced (IIIA–IIIB). The seven-level distribution was also analyzed. Stage analyses included 287 patients; two patients with missing stage were excluded. Staging records for all 289 patients were reviewed, and staging consistency was reassessed. The differences recorded for A08, A11, and A16 reflected disease progression over successive surgical episodes. Total-registry stages were used for A08 (IIC), A11 (IIIB), and A16 (IIA). The eight combinations of their alternative stages and exclusion of these three cases were assessed separately (Supplementary Table S1). Clinical statistical analyses are detailed in Supplementary Table S2.

The detailed subgroup was reviewed for sex, symptoms, stage, recurrence, operations, radiotherapy, death, records of malignant transformation, Ki-67 labeling, and colorectal or rectal cancer history. Recurrence and death were recorded when documented; entries without event documentation were labeled “not documented.” Intervals to death were measured from the first recorded institutional operation. The final follow-up cutoff was July 2026. Survival outcomes were not estimated. Pathology-slide records and database transformation entries were extracted separately. No central pathology review was performed. Other incompletely recorded clinical variables were excluded from comparative analyses.

### 2.3. Preoperative Imaging and Surgical Planning

**Figure 1:**
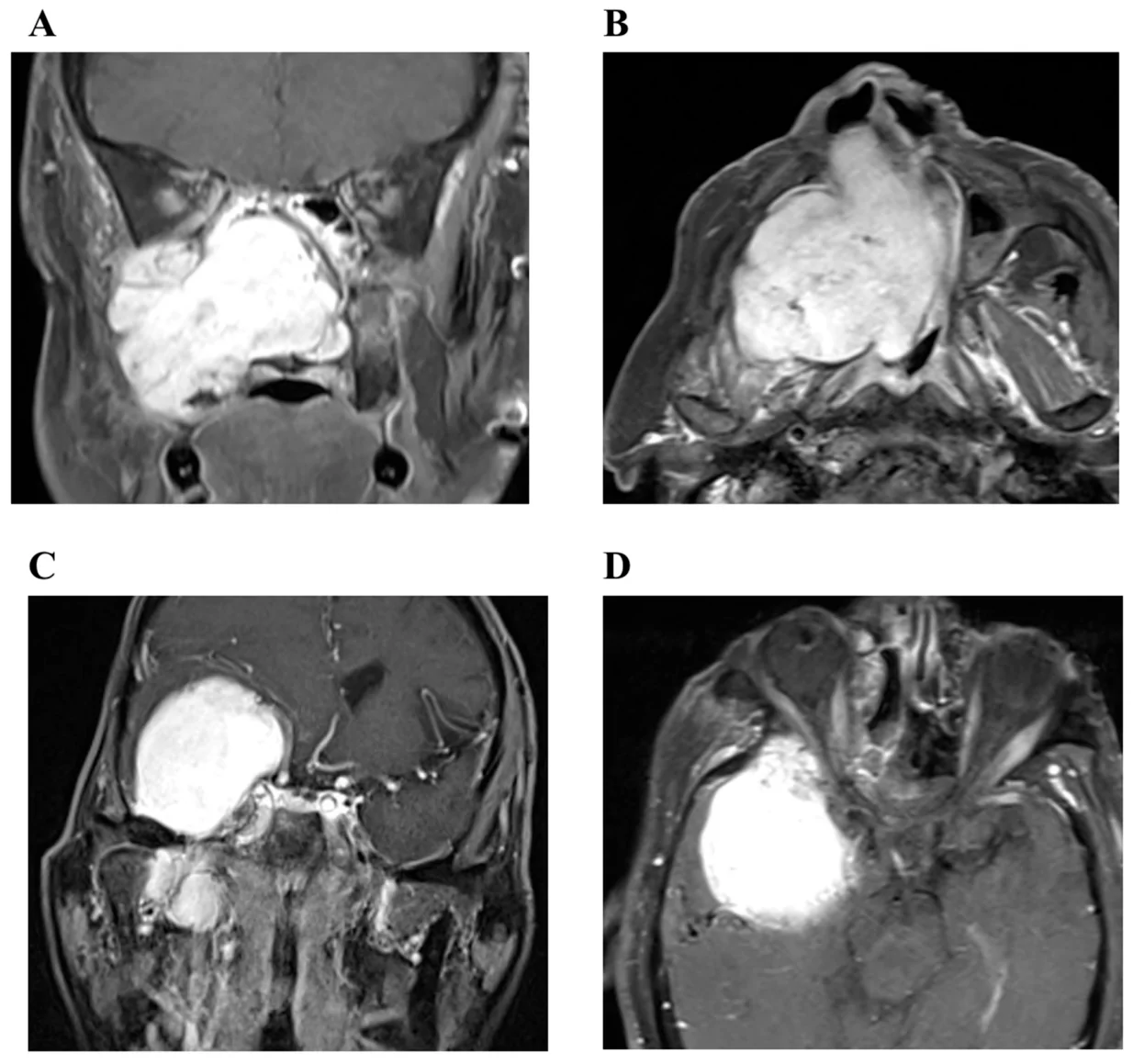
Longitudinal contrast-enhanced MRI of case A13, a 71-year-old man with recurrent stage IIIB nasopharyngeal angiofibroma. (**A**,**B**) Preoperative coronal and axial fat-suppressed T1-weighted images show a large, lobulated, intensely and heterogeneously enhancing mass centered in the right posterior nasal cavity and nasopharynx, with extension through the sphenopalatine region into the pterygopalatine fossa, medial infratemporal fossa, and central skull base. (**C**,**D**) Sagittal and axial contrast-enhanced fat-suppressed T1-weighted images obtained after the third operation show persistent or recurrent enhancing tumor with skull-base destruction and intracranial extension. The serial images illustrate treatment-refractory disease after multiple operations.

Preoperative contrast-enhanced magnetic resonance imaging (MRI) was used to define tumor origin, routes of extension, skull-base or intracranial involvement, vascular flow voids, and the relationship to major vessels; postoperative MRI was used to assess residual disease [13]. For institutional surgical planning, close internal carotid artery (ICA) involvement was flagged when the shortest tumor-to-ICA distance was <1.8 mm on contrast-enhanced MRI. This threshold was derived from our surgical staging work in recurrent nasopharyngeal carcinoma and was used as a pragmatic risk indicator rather than as a validated criterion for ICA wall invasion in NA [14]. Advanced or vessel-adjacent tumors were reviewed with the neurointerventional team. Balloon occlusion testing and ICA pretreatment were considered only when planned resection might require parent-artery sacrifice. The cited experience with ICA pretreatment concerns recurrent nasopharyngeal carcinoma and is used here as a technical reference, not as validation in NA [15]. Angiography and embolization of dominant tumor feeders were planned case by case according to vascular anatomy [16,17]. Because ICA distance, balloon occlusion testing, and embolization were not captured uniformly in the clinical database, they were not included in the comparative analyses. The operative approach was selected according to tumor location and extent. Representative preoperative and postoperative MRI findings are shown in Figure 1.

Radkowski stage was recorded throughout the study period. University of Pittsburgh Medical Center (UPMC) staging also requires information on skull-base extension routes and residual vascularity after embolization [18]. These fields were incomplete in the database, so UPMC stage was not assigned.

### 2.4. Tumor–Normal Whole-Genome Sequencing

The genomic component was an annotation-level review of one tumor–blood pair, based on the supplied small-variant summaries and supplementary record tables. We summarized record counts, substitution classes, variant allele fractions, and functional annotations while retaining the distinction between unique chromosome–position–reference–alternate records and gene-expanded events. PASS was treated as the filtering label reported in the source data, rather than as evidence of independent somatic validation. The coding/splice-selected subset contained 2714 records, although its complete selection criteria were not documented. To preserve the relationship between the summaries and their source records, gene annotations, breakend pairs, altered copy-number segments, and chromosome-Y records are provided in Supplementary Tables S3, S4, S5, and S6, respectively, with functional-category counts and denominators reconciled in Supplementary Table S7.

Copy-number and structural-variant findings were likewise summarized from the available annotation tables. Because the source material did not support raw-read reanalysis or a validated assessment of somatic status and purity/ploidy, these analyses were not performed, and interpretation was limited to the reported annotations rather than tumor mutational burden, mutational signatures, or whole-chromosome Y dosage. Supplementary Table S8 distinguishes the sequencing information available for review from the analyses that could not be assessed or were not performed. The comparison with published exome findings is presented in Supplementary Table S9. For consistency across the annotation tables, gene symbols were checked against HUGO Gene Nomenclature Committee (HGNC) records; the mapping between original and current symbols is retained in Supplementary Table S10. Coding-length and background-rate normalization were unavailable, so gene counts were treated as descriptive source observations rather than a ranking of biological importance.

### 2.5. Statistical Analysis

Each patient contributed one observation. Age is reported as the median and range. Categorical variables are reported as counts and percentages, using the number of patients with available data as the denominator. Two-sided Fisher–Freeman–Halton tests compared stage distributions between age groups, and a Cochran–Armitage test assessed the ordinal stage trend. The post hoc IA-or-IIIB comparison used Fisher’s exact test, an odds ratio with an exact 95% confidence interval, and Bonferroni corrections for 21 and 63 groupings.

Continuous-age analyses, alternative age thresholds, and sensitivity analyses of stage assignments are detailed in Supplementary Table S2. These additional analyses were exploratory. Tests were two-sided, with a nominal significance threshold of *p* < 0.05. Analyses used Python 3.12.14. Software versions, calculation settings, and reproducible code are provided in the Supplementary data package.

## 3. Results

### 3.1. Cohort Assembly and Frequency of Adult Disease

**Figure 2:**
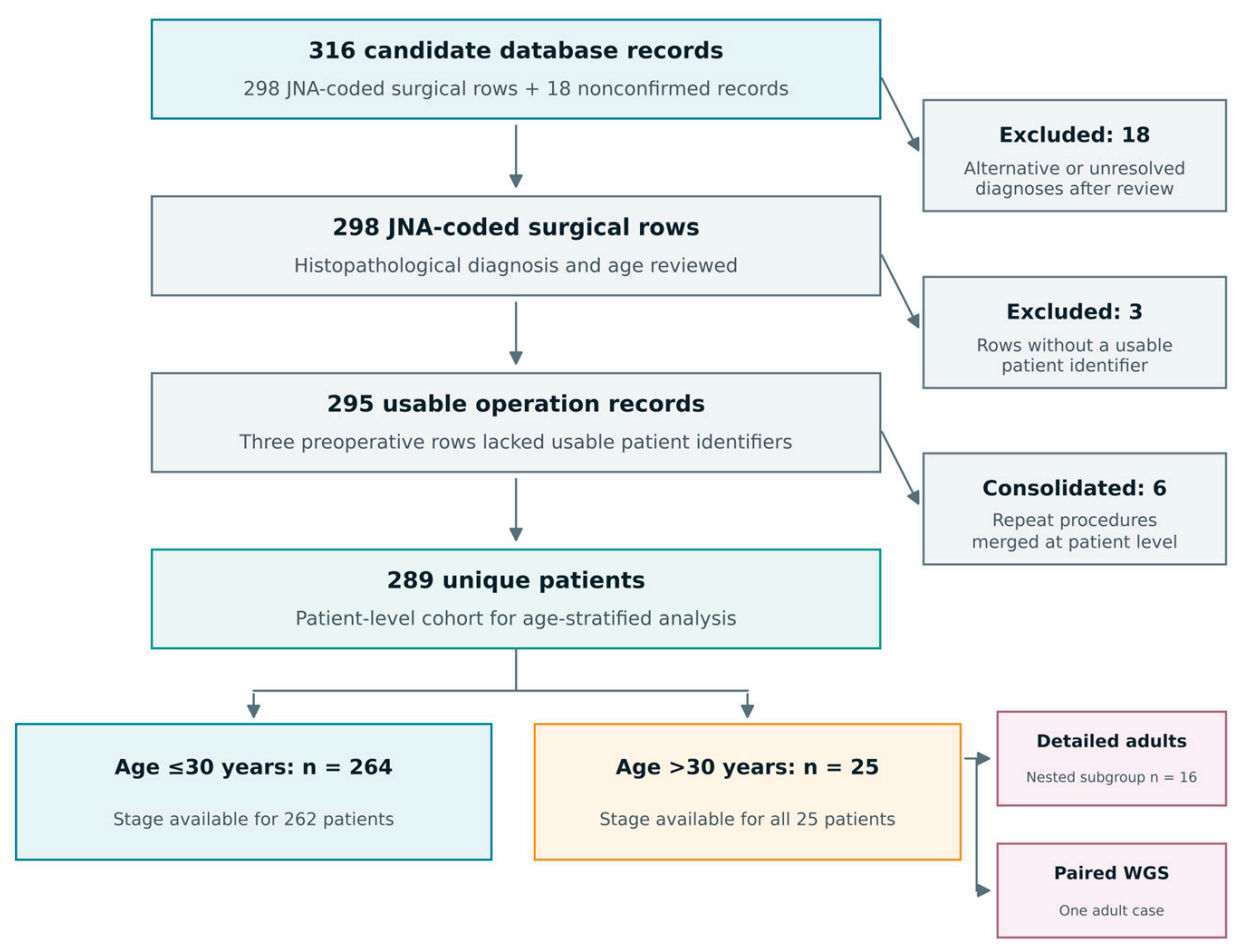
Cohort selection and nested analytical sets from a single institutional clinical database. The database query identified 298 JNA-coded surgical rows and 18 records with diagnoses other than confirmed NA. The latter were excluded after diagnostic review. Three JNA-coded rows without a usable patient identifier were excluded, and six repeat-procedure rows were consolidated, leaving 289 unique patients. The primary age-stage comparison included 264 patients aged ≤ 30 years and 25 aged > 30 years; stage was available for 262 and 25 patients, respectively. Detailed clinical phenotyping was performed in a nested subgroup of 16 adults, including one patient with paired tumor–blood WGS.

**Figure 3:**
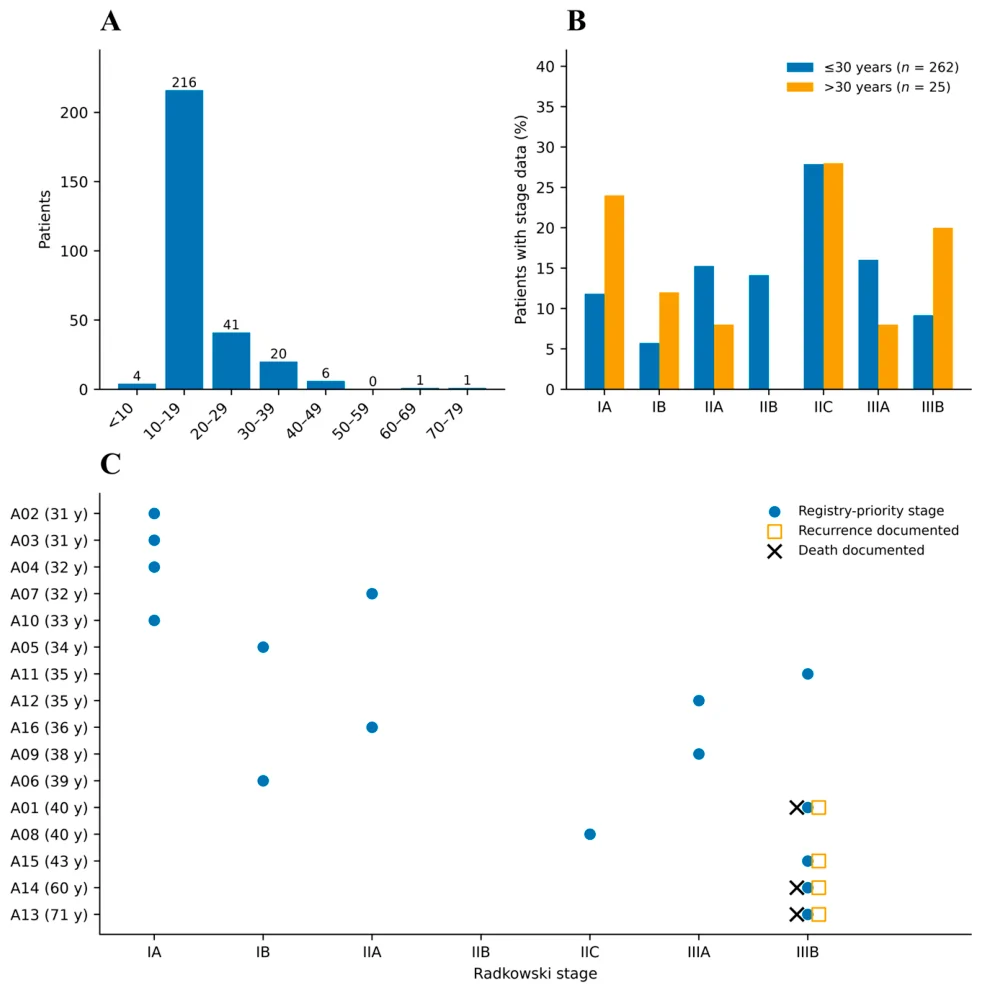
Age and recorded stage. (**A**) Linear-axis age distribution in 289 unique patients, including the empty 50–59-year band. (**B**) Stage proportions among 262 patients aged ≤ 30 years and 25 aged > 30 years; exact comparisons are given in Table 1. (**C**) Registry-priority stage in 16 adults, labeled by case. Recurrence and death markers indicate affirmative documentation; their absence does not establish event-free follow-up. The different stages recorded for A08, A11, and A16 reflected progression over successive surgical episodes. In panel C, filled circles indicate stage, open squares indicate documented recurrence, and crosses indicate documented death. Case labels include age in years.

The database query identified 316 candidate records, comprising 298 JNA-coded surgical rows and 18 records with diagnoses other than confirmed NA. Excluding the 18 nonconfirmed records and three JNA-coded rows without usable identifiers left 295 usable operation records; consolidating six repeat-procedure rows then yielded 289 unique patients (Figure 2). The cohort had a median age of 16 years (range, 8–71 years) and was concentrated in adolescence: 216/289 patients (74.7%) were aged 10–19 years. Outside this age band, 4 patients were aged <10 years, 41 patients were aged 20–29 years, 20 were aged 30–39 years, 6 were aged 40–49 years, and 1 each was aged 60–69 and 70–79 years. The 30–39-year band included three patients aged exactly 30 years, who remained in the ≤30-year reference group (Figure 3A).

**Table 1:** Age and Radkowski stage in the patient-level cohort.

Characteristic	Age ≤ 30 Years	Age > 30 Years	Comparison
Patients, *n*	264	25	-
Age, median (range), years	16 (8–30)	35 (31–71)	Descriptive
Stage available, *n*	262	25	287 total
Radkowski IA, *n* (%)	31 (11.8)	6 (24.0)	Seven-level exact *p* = 0.0367
Radkowski IB, *n* (%)	15 (5.7)	3 (12.0)	-
Radkowski IIA, *n* (%)	40 (15.3)	2 (8.0)	-
Radkowski IIB, *n* (%)	37 (14.1)	0 (0.0)	-
Radkowski IIC, *n* (%)	73 (27.9)	7 (28.0)	-
Radkowski IIIA, *n* (%)	42 (16.0)	2 (8.0)	-
Radkowski IIIB, *n* (%)	24 (9.2)	5 (20.0)	-
Low-stage (IA–IB), *n* (%)	46 (17.6)	9 (36.0)	Three-band exact *p* = 0.0493
Intermediate-stage (IIA–IIC), *n* (%)	150 (57.3)	9 (36.0)	-
Advanced-stage (IIIA–IIIB), *n* (%)	66 (25.2)	7 (28.0)	-
Extreme stage (IA or IIIB), *n* (%)	55 (21.0)	11 (44.0)	Post hoc Fisher *p* = 0.0133; OR 2.94 (95% CI 1.14–7.43); Bonferroni *p* = 0.2800/0.8399

Note: Stage percentages are calculated among 262 patients aged ≤ 30 years and 25 aged > 30 years with available stage data. Fisher–Freeman–Halton tests compare the complete stage distributions, not individual stage rows. The seven-level comparison used 2,000,000 Monte Carlo samples. The post hoc IA-or-IIIB comparison includes Bonferroni-adjusted *p* values for 21 and 63 groupings, respectively. Other secondary and sensitivity *p* values are unadjusted. A dash indicates that no row-specific comparison was performed. CI, confidence interval; OR, odds ratio.

Using the prespecified age threshold, 25 patients (8.7%) were older than 30 years and 264 were aged ≤ 30 years. Their median ages were 35 years (range, 31–71 years) and 16 years (range, 8–30 years), respectively. All 16 patients with detailed adult records were male; however, the absence of a consistent sex field in the full patient-level dataset precluded sex-specific analysis of the cohort.

### 3.2. Age-Stratified Radkowski Stage

Stage data were available for all 25 adults and 262 of 264 reference patients. Adults had a higher proportion of low-stage tumors and a lower proportion of intermediate-stage tumors, whereas the proportion with advanced disease was similar between groups. Specifically, low-stage (IA–IB), intermediate-stage (IIA–IIC), and advanced-stage (IIIA–IIIB) disease accounted for 9/25 (36.0%), 9/25 (36.0%), and 7/25 (28.0%) adult cases, compared with 46/262 (17.6%), 150/262 (57.3%), and 66/262 (25.2%) reference cases. The complete seven-level distribution and exact comparisons are shown in Figure 3B and Table 1.

This difference in the overall distribution was supported by both the three-band comparison (*p* = 0.0493) and the seven-level comparison (*p* = 0.0367). It did not, however, represent a consistent shift toward either lower or higher stage with age: the ordinal trend test was not significant (*p* = 0.5088), and continuous-age analysis indicated a nonlinear association with stage (*p* = 1.59 × 10^−5^; Supplementary Table S2).

Sensitivity analyses examined whether the findings depended on the stages recorded for A08, A11, and A16 at successive surgical episodes. Across the eight combinations of their recorded stages, the three-band *p* value ranged from 0.0061 to 0.0493; excluding these patients yielded *p* = 0.0196 (*n* = 284). Analyses using alternative age thresholds produced *p* values ranging from 0.0120 to 0.3117, indicating that the comparison also depended on the chosen age threshold (Supplementary Table S2).

An exploratory post hoc comparison focused on the combined IA-or-IIIB category, which included 11/25 adults and 55/262 reference patients (OR, 2.94; exact 95% CI, 1.14–7.43; Fisher *p* = 0.0133). This comparison was not significant after correction for multiple testing: the Bonferroni-adjusted *p* values were 0.2800 for 21 groupings and 0.8399 for 63 dichotomizations.

### 3.3. Detailed Adult Clinical Subgroup

The nested subgroup of 16 adults provided the detailed clinical context for the cohort-level stage comparison. Their median age was 35.5 years (range, 31–71 years), with six patients having low-stage, three intermediate-stage, and seven advanced-stage disease. The individual stages were IA in 4 patients, IB in 2, IIA in 2, IIC in 1, IIIA in 2, and IIIB in 5. Figure 3C summarizes their ages, stages, and documented outcomes, and Table 2 presents the corresponding patient-level findings.

Within this subgroup, documented adverse outcomes were concentrated among the five patients with stage IIIB disease, who accounted for all four recorded recurrences and all three deaths. The patients with recurrence were aged 40, 43, 60, and 71 years; recorded operation counts were available for three of them and were 3, 5, and 5, and three recurrent patients received radiotherapy. Death occurred 4.5, 8.2, and 8.7 years after the first institutional operation (Table 2). The persistent disease in A13 was illustrated by MRI after the third operation, which showed enhancing tumor with skull-base destruction and intracranial extension (Figure 1). Symptom documentation was less complete: only three patients had recorded symptoms, including visual impairment in all three, hearing impairment in two, and epistaxis in one.

The records of malignant transformation differed in their pathological support. A01, a 40-year-old man with recurrent stage IIIB disease, had a prior pathology-slide record describing focal malignant transformation and a Ki-67 index of approximately 50%; he underwent three recorded operations and died 8.2 years after the first institutional operation. In contrast, the transformation entries for A15 (43 years) and A13 (71 years) were confined to the database and lacked accompanying pathological documentation.

A separate finding in the clinical histories was colorectal or rectal cancer in three men aged 32, 34, and 39 years at the index NA operation, whose NA stages were IIA, IB, and IB, respectively. The records did not include the dates of colorectal cancer diagnosis, polyp burden, family history, or germline testing results, limiting further characterization of this association.

### 3.4. Exploratory Tumor–Normal WGS Annotation Review

**Figure 4:**
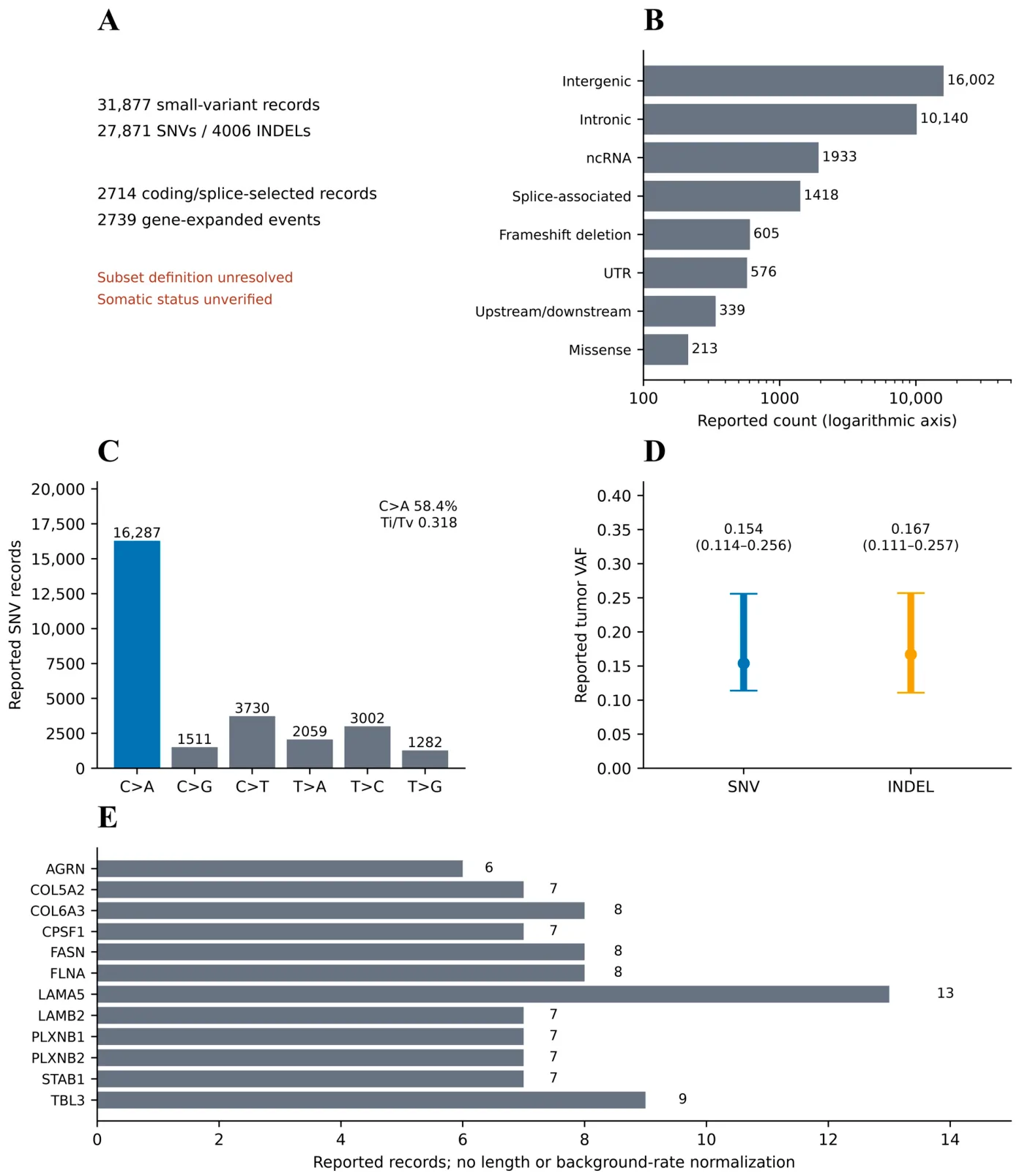
Small-variant annotation summaries from one tumor–blood pair. (**A**) Total small-variant and coding/splice-selected counts. (**B**) Eight functional categories from the whole annotation summary, displayed on a logarithmic count axis. (**C**) Six-class SNV spectrum and transition/transversion ratio. (**D**) VAF medians (points) and interquartile ranges (vertical bars), with blue indicating SNVs and orange indicating INDELs. (**E**) Counts for the 12 previously listed genes, arranged alphabetically; coding-length and background-rate normalization were unavailable. The source annotations have not undergone independent somatic validation; available information on subset selection is provided in Supplementary Table S7. SNV, single-nucleotide variant; INDEL, insertion/deletion; ncRNA, noncoding RNA; UTR, untranslated region; Ti/Tv, transition/transversion ratio; VAF, variant allele fraction.

The single tumor–blood pair yielded a source summary of 31,877 PASS-labeled small-variant records, comprising 27,871 single-nucleotide variants (SNVs) and 4006 insertions/deletions (INDELs). The reported median tumor variant allele fraction (VAF) was 0.156 (interquartile range, 0.114–0.256). Within the SNV summary, C>A substitutions predominated, accounting for 16,287/27,871 records (58.4%), with a transition/transversion ratio of 0.318. The coding/splice-selected subset was reported separately as 2714 distinct records and 2739 gene-expanded events. Figure 4 brings together these counts, the functional categories, substitution spectrum, VAF summaries, and gene counts; the denominators underlying the functional categories are detailed in Supplementary Table S7. The gene counts are descriptive because coding-length and background-rate normalization were unavailable.

**Figure 5:**
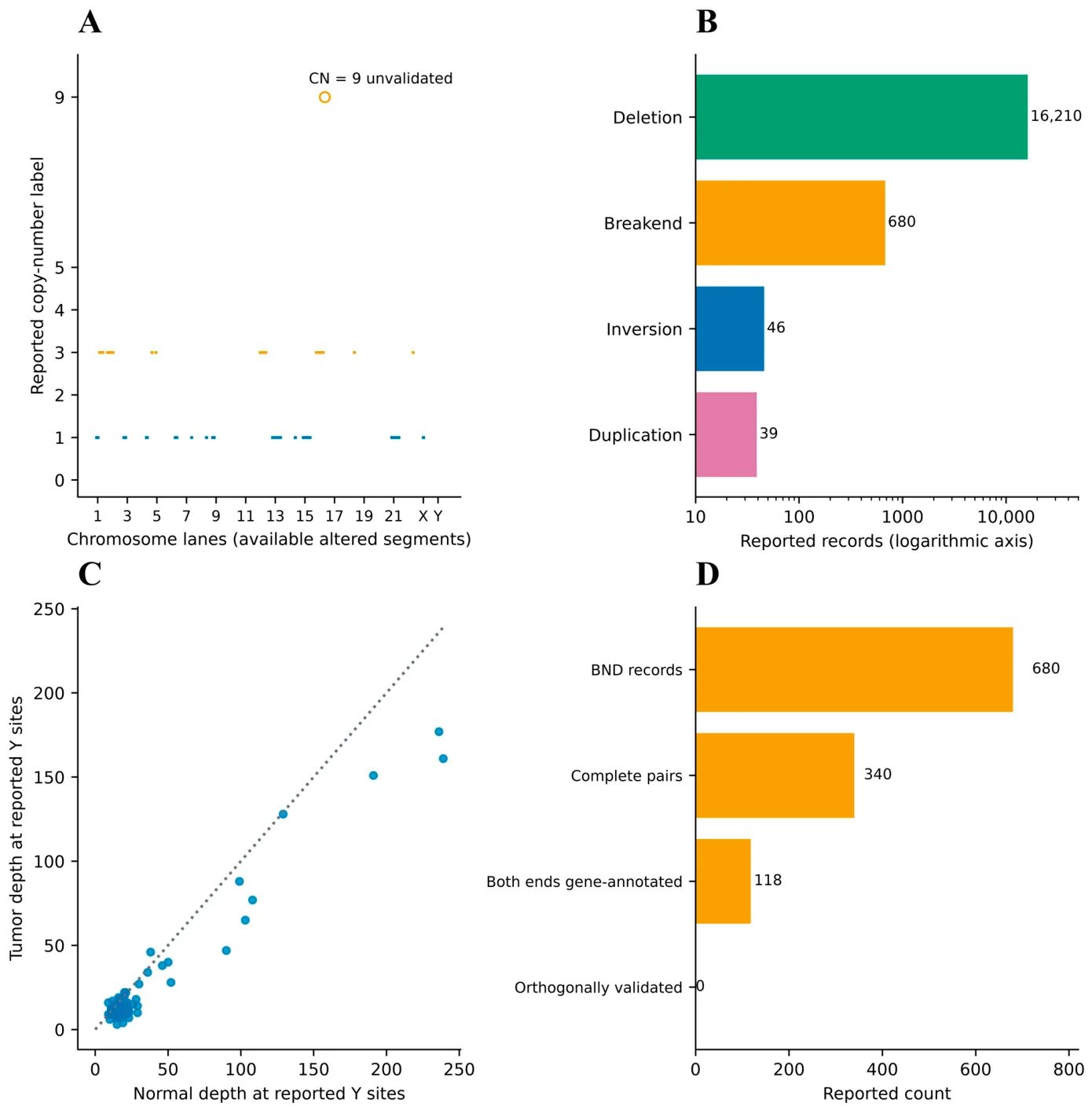
Copy-number and structural-variant annotations. (**A**) The 84 altered segments in Supplementary Table S5, positioned within equal-width chromosome lanes using source coordinates. Orange and blue segments denote source copy-number labels >2 and ≤2, respectively; the colors do not establish biological gain or loss, particularly on sex chromosomes. The open orange circle marks the chromosome-16 segment labeled copy number 9, displayed at its reported value. (**B**) Structural-variant counts on a logarithmic axis. (**C**) Each blue point represents the paired tumor and normal read depths at a selected chromosome-Y site from Supplementary Table S6; the gray dotted diagonal denotes equal depths. (**D**) Breakend-record, mate-pair, and gene-annotation counts. No rearrangements underwent orthogonal validation. The source reference assembly was not provided. Selected-site depths do not measure chromosome-wide copy number. CN, copy number; BND, breakend.

The copy-number and structural-variant summaries described additional features of the same source annotations (Figure 5; Table 3). Of 238 copy-number segments, 51 were labeled gains and 33 losses, together spanning 435.55 Mb. These 84 altered segments are listed in Supplementary Table S5 and include a chromosome-16 segment labeled copy number 9. The structural-variant summary contained 16,975 PASS-labeled records: 16,210 deletions, 680 breakends, 46 inversions, and 39 duplications. Pairing the breakends yielded 340 pairs, of which 118 had gene annotations at both ends. For chromosome Y, the source table contained 71 small-variant records, all labeled noncoding, and a 100 kb Yq11.21 segment labeled copy number 1 and “loss”; no chromosome-Y structural-variant record appeared in the summary. Together with the small-variant findings in Figure 4, these results describe the available annotation profile without establishing the biological significance of the individual labels.

## 4. Discussion

Adults older than 30 years accounted for 8.7% of this surgically treated NA cohort, extending the clinical picture of a tumor most often encountered during adolescence. Their stage distribution differed from that of younger patients, with more low-stage tumors, fewer intermediate-stage tumors, and a similar proportion of advanced disease (Table 1; Figure 3). This pattern should not be interpreted as a uniform increase or decrease in severity with age: the ordinal trend test was not significant, the continuous-age analysis indicated a nonlinear association (Supplementary Table S2), and the exploratory IA-or-IIIB comparison did not remain significant after Bonferroni correction. In conjunction with published adult presentations [6,7] and recurrence after earlier treatment [8], these findings show that NA remains clinically relevant beyond adolescence while emphasizing the heterogeneity of adult disease.

That heterogeneity was also evident in the detailed adult subgroup. Although some patients had limited-stage tumors, advanced disease was accompanied by repeated operations, recurrence, and death; the three recorded deaths occurred 4.5–8.7 years after the first institutional operation (Table 2). The serial MRI findings in A13 provide a clinical illustration, showing persistent enhancing tumor with intracranial extension after the third operation (Figure 1). These observations support continued clinical and imaging follow-up [19,20]. Published evidence also shows that residual or recurrent lesions may remain stable or regress over prolonged follow-up [19], so advanced disease should not be assumed to progress uniformly.

Pathological reassessment is a related concern when recurrent tumors change in appearance or behavior. In A01, a prior pathology-slide record described focal malignant transformation, whereas the transformation entries for A13 and A15 lacked accompanying pathological documentation (Table 2). Maintaining this distinction is essential because local invasion, recurrence, and a high Ki-67 index do not by themselves establish a histological diagnosis of malignancy. Reports of transformation in radiation-naive NA further support reassessment of tumors with changes in growth or histological features [2], but the uneven documentation in our cohort does not permit estimation of transformation incidence.

For patients with advanced or recurrent disease, these clinical concerns must be considered alongside the anatomical constraints on surgery. Recent series link advanced stage to intracranial extension, bleeding, and persistence or recurrence, while supporting endoscopic resection when anatomy permits [21,22]. In our workflow, a tumor-to-ICA distance of <1.8 mm served as a planning flag for close vascular proximity; the threshold was derived from recurrent nasopharyngeal carcinoma surgery and is not a validated criterion for ICA wall invasion in NA [14]. Similarly, the cited experience with ICA pretreatment provides a technical context for collateral assessment and vascular planning when parent-artery sacrifice is considered, rather than NA-specific validation [15]. Radkowski stage therefore remained the basis for comparison across this historical cohort, whereas incomplete recording of skull-base extension routes and residual vascularity after embolization precluded assignment of UPMC stage [18].

The colorectal or rectal cancer histories in three patients raise a separate clinical question concerning possible hereditary predisposition (Table 2). NA has been reported as an extracolonic manifestation of familial adenomatous polyposis (FAP) [12], supporting review of colorectal pathology, polyp burden, and family history, with hereditary cancer assessment according to established indications [23]. In this cohort, however, the recorded ages refer to NA surgery, and neither the dates of colorectal cancer diagnosis nor germline results were available. These histories therefore do not establish FAP, and none of the three patients underwent the reported WGS analysis.

Alongside the clinical findings, the single-case WGS review provides a descriptive account of small-variant, copy-number, and structural-variant annotations (Table 3; Figure 4 and Figure 5). Its main interpretive constraint is that the supplied summaries could not be reassessed at the raw-read level or independently validated, as detailed in Section 2.4 and Supplementary Table S8. For example, the predominance of C>A substitutions (58.4% of SNVs) and the transition/transversion ratio of 0.318 cannot be assigned a biological explanation because raw-read and orientation-bias analyses were not performed and the contribution of sequencing artifacts remains unmeasured. The same limitation applies to interpreting gene counts and rearrangements: coding-length and background-rate normalization and a validated callable-sequence denominator were unavailable, and rearrangements lacked orthogonal validation. The reported profile therefore preserves the source observations without establishing driver status or a validated somatic burden.

These constraints also determine how the single-case findings relate to established molecular work in NA. CTNNB1 alterations are well documented in the literature [3,11], but our source data contained neither a validated CTNNB1 exon-3 assessment nor germline APC results; gene-count tables cannot establish the status of either gene. Moreover, the sequenced patient, A14, had no recorded malignant transformation, so this analysis cannot explain the transformation record in A01. The supplied annotations are best viewed as a starting point for studies using validated variants and functional assessment of Wnt/β-catenin, extracellular-matrix, angiogenic, and FGF/FGFR signaling [3,9]. Published work on CD34-defined microvascular density [24] and karyotyping with fluorescence in situ hybridization [25] provides complementary vascular and cytogenetic context, rather than validation of the present annotations.

The chromosome-Y findings likewise require comparison at the level of the genomic targets actually examined. Kumari et al. studied 13 predominantly young patients with advanced NA and five male controls, reporting 28 protein-coding chromosome-Y positions across 14 genes in nine patients [10]. By contrast, the present single-case summary contained 71 noncoding Y annotations; this difference in genomic targets limits direct comparison of the counts (Supplementary Table S9). Our source table also listed a 100 kb Yq11.21 interval (13,550,000–13,650,000) as copy number 1 and labeled it as a “loss.” However, because the reference assembly and the caller’s sex-chromosome baseline were not provided, and copy number 1 is compatible with the normal male haploid state, this interval label alone provides insufficient evidence of whole-chromosome Y loss.

Several features of the study limit the inferences that can be drawn from these observations. Although the patient-level cohort comprised 289 unique patients, detailed outcomes were available for only 16 adults selected by record completeness; repeated treatment and adverse events can influence record availability and introduce selection bias. Follow-up closed in July 2026, but incomplete younger-group outcomes prevented age-specific outcome comparisons. The differing levels of pathological support—one prior pathology-slide record and two database entries without accompanying pathology—also restrict interpretation of malignant transformation. Staging records were reviewed for all 289 patients, with a repeat assessment of consistency; the differences in three repeatedly operated patients reflected progression across surgical episodes and were examined in sensitivity analyses. This review improves the traceability of stage assignments but does not remove the limitations of a retrospective single-center series. Finally, the genomic component concerns one case and remains limited to annotation summaries without raw-read reanalysis or experimental validation.

Addressing these limitations will require multicenter cohorts that distinguish symptom-onset age from treatment age and use standardized follow-up with central imaging and pathology review. Consistent tumor–normal DNA and RNA analysis across age groups would then allow the clinical patterns described here to be examined alongside validated molecular findings.

## 5. Conclusions

In this surgical cohort, adults accounted for 8.7% of NA cases and had a different stage distribution from patients aged ≤ 30 years. The detailed adult subgroup included recurrent stage IIIB disease, three recorded deaths, and one prior pathology-slide record of focal malignant transformation. These findings support continued clinical follow-up and pathological reassessment of recurrent adult tumors. The single-case WGS findings are descriptive annotations and do not establish an adult-specific molecular subtype.

## Figures and Tables

**Table 2:** Patient-level clinical characteristics and recorded outcomes in the 16-patient nested adult subgroup.

Case	Age	Sex	Stage	Symptoms	Recurrence	Recorded Operations	Radiotherapy	Death	Death Interval (Years)	Malignant-Transformation Evidence	Ki-67	CRC/Rectal Cancer History
A02	31	Male	IA	NR	ND	NR	No/NR	ND	NA	None recorded	NR	No/NR
A03	31	Male	IA	NR	ND	NR	No/NR	ND	NA	None recorded	NR	No/NR
A04	32	Male	IA	NR	ND	NR	No/NR	ND	NA	None recorded	NR	No/NR
A07	32	Male	IIA	NR	ND	NR	No/NR	ND	NA	None recorded	NR	Yes
A10	33	Male	IA	NR	ND	NR	No/NR	ND	NA	None recorded	NR	No/NR
A05	34	Male	IB	NR	ND	NR	No/NR	ND	NA	None recorded	NR	Yes
A11	35	Male	IIIB	NR	ND	NR	No/NR	ND	NA	None recorded	NR	No/NR
A12	35	Male	IIIA	NR	ND	NR	No/NR	ND	NA	None recorded	NR	No/NR
A16	36	Male	IIA	NR	ND	NR	No/NR	ND	NA	None recorded	NR	No/NR
A09	38	Male	IIIA	NR	ND	NR	No/NR	ND	NA	None recorded	NR	No/NR
A06	39	Male	IB	NR	ND	NR	No/NR	ND	NA	None recorded	NR	Yes
A01	40	Male	IIIB	Left-sided epistaxis, visual decline, and hearing loss	Yes	3	No/NR	Yes	8.2	Focal transformation (prior pathology-slide record)	~50%	No/NR
A08	40	Male	IIC	NR	ND	NR	No/NR	ND	NA	None recorded	NR	No/NR
A15	43	Male	IIIB	Visual and hearing decline	Yes	5	Yes	ND	NA	Database entry; pathology not supplied	NR	No/NR
A14	60	Male	IIIB	NR	Yes	NR	Yes	Yes	8.7	None recorded	NR	No/NR
A13	71	Male	IIIB	Left-eye blindness	Yes	5	Yes	Yes	4.5	Database entry; pathology not supplied	NR	No/NR

Note: Patients are ordered by age at the index operation. ND, not documented; NR, not recorded; NA, not applicable or unavailable; CRC, colorectal cancer. ND denotes missing event documentation and does not establish event-free follow-up. No/NR retains the source category combining negative and unrecorded entries. Alternative stages for A08, A11, and A16 are listed in Supplementary Table S1. Intervals to death are measured from the first recorded institutional operation. The source of each transformation entry is specified: a prior pathology-slide record for A01 and database entries without accompanying pathological documentation for A13/A15.

**Table 3:** WGS annotation summary.

Category	Metric	Value
Small variants	Reported PASS-labeled unique SNV/INDEL records	31,877
Small variants	SNVs/INDELs	27,871/4006
Small variants	Reported coding/splice-selected records	2714
Small variants	Reported gene-expanded events	2739
Small variants	Tumor VAF, median (IQR)	0.156 (0.114–0.256)
Copy number	Segments/gains/losses	238/51/33
Copy number	Altered span	435.55 Mb
Structural variants	PASS records	16,975
Structural variants	Deletion/breakend/inversion/duplication	16,210/680/46/39
Chromosome Y	Chromosome-Y annotations/coding or splice annotations	71; 0
Chromosome Y	Reported chromosome-Y structural-variant records	0
Breakends	Complete pairs; both breakpoints gene annotated	340; 118
Validation	Orthogonally validated rearrangements	0
Quality control	Transition/transversion ratio	0.318
Chromosome Y	Reported Yq11.21 copy-number label	1 (100 kb)
Interpretation	Somatic burden, driver status, purity, and whole-Y dosage	Not assessed

Note: Values are annotation counts from the source summaries. Independent somatic validation was not performed. PASS denotes the filtering label in the source data. WGS, whole-genome sequencing; SNV, single-nucleotide variant; INDEL, insertion/deletion; IQR, interquartile range; VAF, variant allele fraction. Mb, megabases; kb, kilobases.

## Data Availability

Deidentified clinical data, analysis scripts, and numerical results are provided as supplementary files. Genomic annotations are included in Supplementary Table S3–S10. Raw sequencing reads and filtered VCF files were not deposited; no genomic accession is available.

## Supplementary Materials

The following supporting information can be downloaded at: https://www.mdpi.com/article/10.3390/cancers18193180/s1: Table S1: Stage records across surgical episodes; Table S2: Clinical statistical analyses; Table S3: Previously ranked gene annotations; Table S4: Unvalidated breakend pair annotations; Table S5: Reported altered copy-number segments; Table S6: Chromosome-Y small-variant annotations; Table S7: Functional-category reconciliation; Table S8: Sequencing information and analytical availability; Table S9: Comparison with the published exome study; Table S10: Gene-symbol audit. The accompanying Supplementary Material contains deidentified clinical data, analysis scripts, numerical results, and source annotation tables.
