# Supplementary material for: Nasopharyngeal Angiofibroma in Adults: Age-Stratified Stage Distribution, Clinical Outcomes, and Exploratory Tumor–Normal Genomics
Source: Cancers (Basel). 2026 Oct 2;18(19):3180. doi: 10.3390/cancers18193180 (PMC13664208; doi:10.3390/cancers18193180)
Supplement: Supplementary file 1 [file cancers-18-03180-s001.zip › cancers-4538425-supplementary.pdf]

Supplementary materials

Nasopharyngeal Angiofibroma in Adults: Age-Stratified Stage Distribution, Clinical Outcomes, and Exploratory Tumor-Normal Genomics

Clinical stage-record comparisons and statistical analyses are followed by genomic annotation records. The genomic data comprise source annotations without independent somatic validation.

Supplementary Table S1 Stage records across surgical episodes

Case	Detailed text	Detailed numeric	Total registry	Revision primary	Original resolved
A08	IIIA	IIIA	IIC	IIC	IIIA
A11	IIIB	IIIA	IIIB	IIIB	IIIB
A16	Missing	IA	IIA	IIA	IIA

Staging records for all 289 patients were reviewed, and staging consistency was reassessed. The different stages recorded for A08, A11, and A16 reflected disease progression over successive surgical episodes. Total-registry stages were used consistently in the full cohort and detailed subgroup: A08 IIC, A11 IIIB, and A16 IIA. All eight combinations of the recorded stages were retained in sensitivity analyses.

Supplementary Table S2 Clinical statistical analyses

S2A Original age-group comparisons

Analysis	Result
Three-band Fisher–Freeman–Halton	0.0493
Seven-level Monte Carlo exact	0.0367
95% Monte Carlo uncertainty interval	0.0364–0.0369
Monte Carlo settings	2,000,000 tables; seed 42; probability ordering
Cochran–Armitage trend	Z = -0.661; P = 0.5088
Post hoc Fisher exact	0.0133
Conditional odds ratio and exact 95% CI	2.94 (1.14–7.43)
Bonferroni P for 21 / 63 groupings	0.2800 / 0.8399

S2B Continuous-age models

Intermediate stage is the reference outcome. Age is centered at 16 and expressed in decades. Linear-age global likelihood-ratio P = 0.0178; quadratic-age global P = 4.57e-06; quadratic-versus-linear P = 1.59e-05. Coefficients below are log-relative odds; intervals are Wald 95% intervals. In the quadratic model, the linear coefficient is not a constant age effect. Stage comparisons used probability-ordered Fisher–Freeman–Halton tests: full enumeration for three bands and 2,000,000 fixed-margin Monte Carlo samples for seven levels (seed 42; P = (b + 1)/(B + 1)). The simulation interval for the seven-level P value was 0.0364–0.0369. The ordinal trend test used scores 1–7 for stages IA–IIIB. Analyses used Python 3.12.14, NumPy 2.5.3, pandas 3.0.5, SciPy 1.18.1, and statsmodels 0.15.0.

Linear age

Stage	Term	Coefficient	95% CI	P

Stage	Term	Coefficient	95% CI	P
Low	constant	-1.210	-1.544 to -0.876	1.29e-12
Low	Age / decade	0.520	0.152 to 0.888	0.0056
Advanced	constant	-0.801	-1.087 to -0.516	3.86e-08
Advanced	Age / decade	0.141	-0.254 to 0.536	0.4848

Linear and quadratic age

Stage	Term	Coefficient	95% CI	P
Low	constant	-1.254	-1.617 to -0.892	1.13e-11
Low	Age / decade	1.689	0.697 to 2.680	0.0008
Low	Age²	-0.565	-1.093 to -0.038	0.0357
Advanced	constant	-0.902	-1.209 to -0.594	9.03e-09
Advanced	Age / decade	-0.795	-1.584 to -0.005	0.0485
Advanced	Age²	0.403	0.012 to 0.794	0.0432

S2C Alternative age thresholds

Older group	Older n	Reference n	Three-band P	Trend P
>25	40	247	0.0120	0.0109
>=30	28	259	0.0168	0.1743
>30	25	262	0.0493	0.5088
>35	11	276	0.3117	0.1528
>40	5	282	0.1960	0.0147

Restricting the analysis to age ≤40 years (n = 282) yielded linear global P = 8.28e-05, quadratic global P = 3.47e-05, and quadratic-versus-linear P = 0.0301.

S2D All documented stage alternatives

A08	A11	A16	Three-band P	Post hoc P	Bonf 21	Bonf 63
IIC	IIIB	IIA	0.0493	0.0133	0.2800	0.8399
IIC	IIIB	IA	0.0186	0.0050	0.1059	0.3178
IIC	IIIA	IIA	0.0493	0.0432	0.9062	1.0000
IIC	IIIA	IA	0.0186	0.0133	0.2800	0.8399
IIIA	IIIB	IIA	0.0259	0.0133	0.2800	0.8399
IIIA	IIIB	IA	0.0061	0.0050	0.1059	0.3178
IIIA	IIIA	IIA	0.0259	0.0432	0.9062	1.0000
IIIA	IIIA	IA	0.0061	0.0133	0.2800	0.8399

Excluding the three patients with stage records from different surgical episodes: n = 284; three-band P = 0.0196; linear-age global P = 0.0080; quadratic-age global P = 1.77e-06. All added analyses are exploratory.

Secondary and sensitivity P values are unadjusted except for the two explicitly labeled post hoc Bonferroni corrections. Machine-readable files also contain the trend and age-model results for every stage assignment.

Source: the two deidentified clinical CSVs, the original Supplementary Table S1, and analyze_revision.py.

Supplementary Table S3 Previously ranked gene annotations

The 50 original gene-count records are retained and displayed by current gene symbol, with the original rank preserved as a source locator. Coding-length and background-rate normalization and a validated callable-base denominator were unavailable; neither the counts nor the original ranks should therefore be interpreted as evidence of driver status. Population allele-frequency filtering and independent somatic validation were not performed, and gene-level VAF summaries cannot distinguish common from rare alleles.

Original rank	Original symbol	Current symbol	Reported records	Median VAF	Maximum VAF
44	ACADVL	ACADVL	4	0.169	0.214
45	ADAMTS8	ADAMTS8	4	0.162	0.227
12	AGRN	AGRN	6	0.471	0.538
46	ATP13A1	ATP13A1	4	0.122	0.167
13	B4GALNT4	B4GALNT4	6	0.143	0.231
14	BAIAP3	BAIAP3	6	0.099	0.302
47	BMP1	BMP1	4	0.232	0.278
24	CDH11	CDH11	5	0.160	0.217
25	CELSR2	CELSR2	5	0.222	0.237
48	CLSTN1	CLSTN1	4	0.176	0.208
49	COL4A1	COL4A1	4	0.245	0.559
6	COL5A2	COL5A2	7	0.136	0.431
3	COL6A3	COL6A3	8	0.143	0.295
50	COL7A1	COL7A1	4	0.128	0.192
7	CPSF1	CPSF1	7	0.189	0.390
15	DYNC1H1	DYNC1H1	6	0.136	0.226

Original rank	Original symbol	Current symbol	Reported records	Median VAF	Maximum VAF
16	ESYT1	ESYT1	6	0.134	0.235
4	FASN	FASN	8	0.403	0.559
5	FLNA	FLNA	8	0.567	0.697
27	ILVBL	HACL2	5	0.200	0.333
26	IARS	IARS1	5	0.139	0.324
1	LAMA5	LAMA5	13	0.192	0.326
8	LAMB2	LAMB2	7	0.244	0.373
28	LTBP3	LTBP3	5	0.118	0.237
17	MAN2C1	MAN2C1	6	0.110	0.125
29	MAPK8IP3	MAPK8IP3	5	0.148	0.222
30	MBD1	MBD1	5	0.077	0.218
32	MXRA8	MXRA8	5	0.246	0.444
33	MYH9	MYH9	5	0.167	0.294
34	NISCH	NISCH	5	0.259	0.435
35	OBSL1	OBSL1	5	0.219	0.333
36	PHF1	PHF1	5	0.130	0.214
37	PKD1	PKD1	5	0.226	0.278
18	PLCG1	PLCG1	6	0.104	0.171
9	PLXNB1	PLXNB1	7	0.180	0.350
10	PLXNB2	PLXNB2	7	0.360	0.396

Original rank	Original symbol	Current symbol	Reported records	Median VAF	Maximum VAF
38	POLD1	POLD1	5	0.310	0.324
19	PRRC2A	PRRC2A	6	0.164	0.233
20	RHOT2	RHOT2	6	0.170	0.316
39	SART1	SART1	5	0.133	0.139
21	SBF1	SBF1	6	0.144	0.224
40	SCARB2	SCARB2	5	0.100	0.188
22	SCRIB	SCRIB	6	0.197	0.293
31	MFSD10	SLC75A1	5	0.241	0.263
23	SNRNP200	SNRNP200	6	0.103	0.171
11	STAB1	STAB1	7	0.170	0.269
41	TAF1C	TAF1C	5	0.167	0.211
2	TBL3	TBL3	9	0.154	0.250
42	TLN1	TLN1	5	0.131	0.218
43	TRIM28	TRIM28	5	0.614	0.833

Supplementary Table S4 Unvalidated breakend pair annotations

The table contains 118 paired breakend annotations. Previous gene symbols were mapped to current HGNC symbols, with original identifiers preserved in the source data and gene audit. Mappability/normal-panel filtering, breakpoint-PCR, and RNA confirmation were not performed. Scores are reported as supplied. The reference assembly was not provided.

Rank	Breakend 1	Breakend 2	Gene-pair annotation	Function 1	Function 2	Source score	Min depth	Status
1	chr6:74002281	chr8:109240603	KHDC1--EIF3E	intronic	exonic	99	31	Unvalidated

Rank	Breakend 1	Breakend 2	Gene-pair annotation	Function 1	Function 2	Source score	Min depth	Status
2	chr2:9770287	chr4:164853960	YWHAQ--MARCHF1	splicing	intronic	88	26	Unvalidated
3	chr10:127355838	chr16:30080905	TEX36--ALDOA	intronic	exonic	80	33	Unvalidated
4	chr10:127355966	chr16:30080616	TEX36--ALDOA	intronic	intronic	80	35	Unvalidated
5	chr9:34319009	chr11:75278001	KIF24--SERPINH1	intronic	exonic	80	23	Unvalidated
6	chr11:122930728	chrX:114010763	HSPA8--HTR2C	exonic	intronic	76	33	Unvalidated
7	chr5:176693282	chr19:50187183	NSD1--PRMT1	intronic	splicing	76	26	Unvalidated
8	chr2:232323097	chr9:139708444	NUCLEOLIN--RABL6	exonic	intronic	72	34	Unvalidated
9	chr1:202408210	chr19:18649026	PPP1R12B--FKBP8	intronic	exonic	69	25	Unvalidated
10	chr4:77880277	chr9:113013559	SEPTIN11--TXN	intronic	intronic	66	35	Unvalidated
11	chr12:120900021	chr15:83360550	GATC--CPEB1-AS1	UTR3	ncRNA_intronic	65	28	Unvalidated
12	chr17:74222517	chr22:39917759	RNF157--ATF4	intronic	intronic	64	30	Unvalidated
13	chr1:197191735	chrX:40456779	CRB1--ATP6AP2	intronic	exonic	64	15	Unvalidated
14	chr1:8925609	chr17:4858735	ENO1--ENO3	intronic	exonic	63	35	Unvalidated
15	chr1:93301709	chr8:42199757	DIPK1A--POLB;RPL5--POLB	intronic	intronic	62	44	Unvalidated
16	chr2:96940901	chr17:62747330	SNRNP200--ARHGAP27P1-BPTFP1-KPNA2P3	intronic	ncRNA_intronic	61	24	Unvalidated
17	chr2:203044601	chr7:42957275	KIAA2012--PSMA2	intronic	exonic	60	25	Unvalidated
18	chr3:154039069	chr6:118410872	DHX36--LOC105377967	intronic	ncRNA_intronic	60	42	Unvalidated
19	chr1:32696714	chr7:109599343	EIF3I--EIF3IP1	splicing	ncRNA_exonic	58	27	Unvalidated
20	chr3:196904294	chr9:33568768	DLG1--ANKRD18B	intronic	exonic	58	44	Unvalidated
21	chr1:153825735	chr9:19126348	GATAD2B--PLIN2	intronic	intronic	55	41	Unvalidated
22	chr6:116579625	chr19:58905981	DSE--RPS5	UTR5	intronic	55	19	Unvalidated

Rank	Breakend 1	Breakend 2	Gene-pair annotation	Function 1	Function 2	Source score	Min depth	Status
23	chr1:240306606	chr3:184025349	FMN2--PSMD2	intronic	intronic	54	38	Unvalidated
24	chr1:72740798	chr10:5808098	NEGR1--GDI2	intronic	exonic	54	40	Unvalidated
25	chr16:28855819	chr17:25410208	TUFM--LOC105371703	exonic	ncRNA_intronic	53	35	Unvalidated
26	chr1:146790806	chr9:114359527	NBPF19--PTGR1	intronic	intronic	53	43	Unvalidated
27	chr1:160098357	chr19:42489016	ATP1A2--ATP1A3	intronic	intronic	53	25	Unvalidated
28	chr1:211035449	chr11:9430031	KCNH1--IPO7	intronic	splicing	52	35	Unvalidated
29	chr1:27651828	chr17:79478218	ACTG1P20--ACTG1	ncRNA_exonic	exonic	52	40	Unvalidated
30	chr2:110942319	chr10:104243917	NPHP1--ACTR1A	intronic	exonic	52	46	Unvalidated
31	chr3:133969265	chr17:4125595	RYK--ANKFY1	splicing	intronic	52	18	Unvalidated
32	chr5:137847282	chr7:151199972	ETF1--RHEB	exonic	intronic	52	39	Unvalidated
33	chr9:19027398	chr11:47445827	SAXO1--PSMC3	intronic	intronic	52	31	Unvalidated
34	chr10:47595301	chr20:32878353	ANTXRLP1--AHCY	ncRNA_intronic	splicing	51	31	Unvalidated
35	chr11:43920145	chr17:75202308	SEC14L1P1--SEC14L1	ncRNA_exonic	splicing	51	29	Unvalidated
36	chr1:240308355	chr3:184018004	FMN2--PSMD2	intronic	intronic	51	39	Unvalidated
37	chr2:198352807	chr5:21884172	HSPD1--CDH12	intronic	intronic	51	40	Unvalidated
38	chr16:28855320	chr17:25410156	TUFM--LOC105371703	exonic	ncRNA_intronic	50	30	Unvalidated
39	chr1:238090650	chr17:79478691	LOC100130331--ACTG1	ncRNA_exonic	intronic	50	37	Unvalidated
40	chr1:27652131	chr17:79478052	ACTG1P20--ACTG1	ncRNA_exonic	exonic	50	25	Unvalidated
41	chr3:111289982	chr19:45385724	CD96--NECTIN2	intronic	intronic	50	34	Unvalidated
42	chr11:43352647	chr19:6987048	API5--ADGRE4P	intronic	ncRNA_intronic	49	30	Unvalidated
43	chr11:43918910	chr17:75138912	SEC14L1P1--SEC14L1	ncRNA_exonic	intronic	49	34	Unvalidated

Rank	Breakend 1	Breakend 2	Gene-pair annotation	Function 1	Function 2	Source score	Min depth	Status
44	chr1:153635412	chr3:65673762	ILF2--MAGI1	intronic	intronic	49	37	Unvalidated
45	chr1:240307486	chr3:184021033	FMN2--PSMD2	intronic	intronic	49	35	Unvalidated
46	chr6:116580020	chr19:58904570	DSE--RPS5	UTR5	intronic	49	30	Unvalidated
47	chr6:129250185	chr7:130144730	LAMA2--MEST	intronic	intronic	49	35	Unvalidated
48	chr11:122889184	chr19:47700434	LOC341056--SAE1	ncRNA_exonic	intronic	48	38	Unvalidated
49	chr16:4862352	chr20:48447112	GLYR1--SLC9A8	intronic	intronic	48	27	Unvalidated
50	chr10:102286155	chr17:57962821	NDUFB8--TUBD1	splicing	intronic	47	23	Unvalidated
51	chr1:240306374	chr3:184025895	FMN2--PSMD2	intronic	intronic	47	34	Unvalidated
52	chr1:93302959	chr15:25154321	DIPK1A--SNHG14;RPL5--SNHG14	intronic	ncRNA_intronic	47	33	Unvalidated
53	chr2:47389065	chr10:71923815	CALM2--SAR1A	intronic	intronic	47	40	Unvalidated
54	chr12:120901971	chr15:83360648	SRSF9--CPEB1-AS1	intronic	ncRNA_intronic	46	32	Unvalidated
55	chr12:6994578	chr20:17587678	DSTNP2--DSTN	ncRNA_exonic	splicing	46	29	Unvalidated
56	chr1:220427926	chr15:79189408	RAB3GAP2--MORF4L1	intronic	exonic	46	30	Unvalidated
57	chr3:101298878	chr13:48902568	PCNP--RB1	intronic	intronic	46	32	Unvalidated
58	chr12:10875397	chr16:31580674	YBX3--YBX3P1	intronic	ncRNA_exonic	45	20	Unvalidated
59	chr1:160905705	chr7:44612489	LOC101928372--DDX56	ncRNA_intronic	exonic	45	37	Unvalidated
60	chr1:17739983	chr11:62140263	RCC2--ASRGL1	intronic	intronic	45	31	Unvalidated
61	chr5:108230081	chr6:31765741	FER--LSM2	intronic	intronic	45	31	Unvalidated
62	chr5:176693344	chr19:50185322	NSD1--PRMT1	intronic	exonic	44	24	Unvalidated
63	chr7:128696671	chr12:6979105	TPI1P2--TPI1	ncRNA_exonic	intronic	44	39	Unvalidated
64	chr12:10865809	chr16:31580042	YBX3--YBX3P1	splicing	ncRNA_exonic	43	27	Unvalidated

Rank	Breakend 1	Breakend 2	Gene-pair annotation	Function 1	Function 2	Source score	Min depth	Status
65	chr1:92591210	chr17:66520053	BTBD8--PRKAR1A	intronic	intronic	43	39	Unvalidated
66	chr2:98823389	chr12:57037111	VWA3B--ATP5F1B	intronic	intronic	43	40	Unvalidated
67	chr5:145838492	chr6:31611660	TCERG1--BAG6	exonic	intronic	43	26	Unvalidated
68	chr5:173043202	chr18:54814595	BOD1--BOD1L2	splicing	exonic	43	26	Unvalidated
69	chr8:75515453	chr12:53873226	MIR2052HG--PCBP2	ncRNA_intronic	exonic	43	34	Unvalidated
70	chr11:66529604	chr17:5341389	TOP6BL--C1QBP	intronic	intronic	42	32	Unvalidated
71	chr12:10854494	chr16:31579893	YBX3--YBX3P1	intronic	ncRNA_exonic	42	20	Unvalidated
72	chr1:160105478	chr19:42480417	ATP1A2--ATP1A3	intronic	intronic	42	16	Unvalidated
73	chr1:171308440	chr20:39742792	TOP1P1--TOP1	ncRNA_exonic	exonic	42	29	Unvalidated
74	chr2:11397499	chr3:88108641	ROCK2--ZNF654	intronic	exonic	42	21	Unvalidated
75	chr5:141511904	chr12:18511621	NDFIP1--PIK3C2G	exonic	intronic	42	38	Unvalidated
76	chr11:122888501	chr19:47646939	LOC341056--SAE1	ncRNA_exonic	intronic	41	30	Unvalidated
77	chr12:104326510	chr15:99798159	HSP90B1--HSP90B2P	intronic	ncRNA_exonic	41	38	Unvalidated
78	chr12:10862476	chr16:31580129	YBX3--YBX3P1	intronic	ncRNA_exonic	41	29	Unvalidated
79	chr3:142546281	chr8:146016920	PCOLCE2--RPL8	intronic	intronic	41	29	Unvalidated
80	chr4:4543480	chr17:28395744	STX18--EFCAB5	intronic	intronic	41	30	Unvalidated
81	chr7:18067429	chrX:106871998	PRPS1L1--PRPS1	UTR5	intronic	41	18	Unvalidated
82	chr12:10862752	chr16:31580098	YBX3--YBX3P1	intronic	ncRNA_exonic	40	34	Unvalidated
83	chr1:202408748	chr19:18643090	PPP1R12B--FKBP8	intronic	intronic	40	21	Unvalidated
84	chr1:227452431	chr3:132395234	CDC42BPA--NPHP3-ACAD11	intronic	ncRNA_intronic	40	29	Unvalidated
85	chr1:27268209	chr2:179319388	NUDC--MIR548N	intronic	ncRNA_intronic	40	24	Unvalidated

Rank	Breakend 1	Breakend 2	Gene-pair annotation	Function 1	Function 2	Source score	Min depth	Status
86	chr11:14539365	chrX:79965414	PSMA1--BRWD3	intronic	intronic	39	20	Unvalidated
87	chr13:30870235	chr19:12911907	KATNAL1--PRDX2	intronic	intronic	39	28	Unvalidated
88	chr3:32794726	chr13:53235705	CNOT10--SUGT1	intronic	exonic	39	23	Unvalidated
89	chr6:90011725	chr16:89999779	GABRR2--TUBB3	intronic	intronic	39	18	Unvalidated
90	chr7:128766818	chr19:4117676	LOC407835--MAP2K2	ncRNA_exonic	intronic	39	20	Unvalidated
91	chr1:888028	chr2:132201370	NOC2L--NOC2LP2	intronic	ncRNA_exonic	38	24	Unvalidated
92	chr11:90016288	chr12:49579778	DISC1FP1--TUBA1A	ncRNA_intronic	splicing	37	25	Unvalidated
93	chr3:100432736	chr6:38555153	TFG--BTBD9	intronic	intronic	37	29	Unvalidated
94	chr3:139213479	chr17:79478053	LOC100507291--ACTG1	ncRNA_intronic	exonic	37	27	Unvalidated
95	chr5:108388077	chr6:121767906	FER--GJA1	intronic	intronic	37	35	Unvalidated
96	chr1:11031222	chr4:15134939	CIROZ--LOC101929095	intronic	ncRNA_intronic	36	29	Unvalidated
97	chr12:10365860	chr15:90892446	GABARAPL1--GABARAPL3	intronic	ncRNA_exonic	35	19	Unvalidated
98	chr12:31944923	chr17:73774880	H3-5--H3-3B	exonic	intronic	35	25	Unvalidated
99	chr1:78557537	chr3:156262230	GIPC2--SSR3	intronic	splicing	35	39	Unvalidated
100	chr1:100679237	chr7:97920342	DBT--BRI3	intronic	intronic	34	19	Unvalidated
101	chr1:58577797	chr5:124047175	DAB1--ZNF608	intronic	intronic	34	33	Unvalidated
102	chr2:152869328	chr19:38270158	CACNB4--ZNF573	intronic	intronic	34	31	Unvalidated
103	chr3:119249864	chr12:77252816	CD80--CSRP2	intronic	intronic	34	36	Unvalidated
104	chr3:169663701	chrX:135292050	LOC100128164--FHL1	ncRNA_exonic	exonic	34	14	Unvalidated
105	chr3:41900741	chr5:172461327	ULK4--ATP6V0E1	intronic	intronic	34	41	Unvalidated
106	chr4:140036439	chr11:64013930	ELF2--PPP1R14B	intronic	exonic	34	20	Unvalidated

Rank	Breakend 1	Breakend 2	Gene-pair annotation	Function 1	Function 2	Source score	Min depth	Status
107	chr11:66529673	chr17:5342131	TOP6BL--C1QBP	intronic	intronic	33	18	Unvalidated
108	chr12:4763680	chr22:36294277	NDUFA9--RBFOX2	intronic	intronic	33	24	Unvalidated
109	chr8:101733594	chr12:64215934	PABPC1--RXYLT1-AS1	intronic	ncRNA_exonic	33	22	Unvalidated
110	chr2:47387946	chr10:71923899	CALM2--SAR1A	splicing	intronic	32	36	Unvalidated
111	chr6:90011692	chr16:89998918	GABRR2--TUBB3	intronic	intronic	32	18	Unvalidated
112	chr7:128767352	chr19:4099408	LOC407835--MAP2K2	ncRNA_exonic	exonic	32	21	Unvalidated
113	chr1:67895724	chr4:79102977	SERBP1--FRAS1	exonic	intronic	31	16	Unvalidated
114	chr3:113604124	chr17:7154502	GRAMD1C--CTDNEP1	intronic	intronic	31	20	Unvalidated
115	chr6:170105402	chrX:154522723	PHF10--CLIC2	exonic	intronic	31	25	Unvalidated
116	chr11:16004303	chr20:36155665	SOX6--BLCAP	intronic	intronic	30	27	Unvalidated
117	chr1:23649972	chr10:21914764	HNRNPR--MLLT10	intronic	intronic	30	38	Unvalidated
118	chr2:136269539	chr5:151166323	ZRANB3--G3BP1	intronic	intronic	30	28	Unvalidated

Supplementary Table S5 Reported altered copy-number segments

The table contains 84 altered segments with a total interval length of 435.55 Mb. Source values include copy number 9 at chromosome 16:33,900,000–34,000,000 and copy number 1, labeled "loss," at Y:13,550,000–13,650,000. Purity-aware resegmentation and clinical CNV classification were not performed. Reference assembly, coordinate conventions, and the sex-chromosome baseline were not supplied.

Chromosome	Start	End	Length, Mb	Predicted copy number	Alteration	Cytoband
1	59,000,000	59,300,000	0.300	1	loss	1p32.2-p32.1
1	89,300,000	104,150,000	14.850	1	loss	1p21.2-p21.1
1	104,300,000	120,550,000	16.250	1	loss	1p13.2-p13.1
1	120,650,000	121,350,000	0.700	1	loss	1p11.2

Chromosome	Start	End	Length, Mb	Predicted copy number	Alteration	Cytoband
1	142,900,000	143,250,000	0.350	4	gain	1q21.1
1	147,700,000	147,750,000	0.050	3	gain	1q21.2
1	155,900,000	156,100,000	0.200	3	gain	1q22
1	156,100,000	156,350,000	0.250	3	gain	1q22
1	156,350,000	169,100,000	12.750	3	gain	1q23.1-q23.2
1	169,100,000	171,400,000	2.300	3	gain	1q24.3-q24.2
1	171,400,000	172,050,000	0.650	3	gain	1q24.3
1	172,050,000	177,250,000	5.200	3	gain	1q25.2-q25.1
1	177,250,000	177,400,000	0.150	3	gain	1q25.2
1	177,400,000	178,250,000	0.850	3	gain	1q25.2
1	178,250,000	178,700,000	0.450	3	gain	1q25.2
1	178,700,000	182,900,000	4.200	3	gain	1q25.3-q25.2
1	182,900,000	183,400,000	0.500	3	gain	1q25.3
1	183,400,000	189,450,000	6.050	3	gain	1q31.1-q25.3
1	189,450,000	200,950,000	11.500	3	gain	1q32.1-q31.2
1	200,950,000	207,700,000	6.750	3	gain	1q32.2-q32.1
1	207,700,000	207,750,000	0.050	1	loss	1q32.2
1	207,750,000	213,150,000	5.400	3	gain	1q32.2-q32.3
1	220,500,000	228,750,000	8.250	3	gain	1q41-q42.12
2	50,000	51,200,000	51.150	3	gain	2p23.2-p21

Chromosome	Start	End	Length, Mb	Predicted copy number	Alteration	Cytoband
2	84,550,000	87,150,000	2.600	3	gain	2p11.2
2	87,550,000	87,650,000	0.100	3	gain	2p11.2
2	95,350,000	95,600,000	0.250	3	gain	2q11.1
2	95,600,000	96,100,000	0.500	3	gain	2q11.1
2	162,100,000	162,150,000	0.050	4	gain	2q24.2
3	19,800,000	44,350,000	24.550	1	loss	3p21.32-p21.31
3	129,750,000	129,800,000	0.050	1	loss	3q22.1
4	103,300,000	114,800,000	11.500	1	loss	4q25-q26
5	50,000	4,350,000	4.300	3	gain	5p15.33
5	46,050,000	46,400,000	0.350	3	gain	5p12-p11
5	112,900,000	113,600,000	0.700	3	gain	5q22.2-q22.3
6	132,150,000	158,500,000	26.350	1	loss	6q25.1-q24.3
7	6,800,000	6,850,000	0.050	1	loss	7p22.1
7	77,700,000	78,500,000	0.800	1	loss	7q21.11
8	112,600,000	112,900,000	0.300	1	loss	8q23.3
9	11,600,000	22,850,000	11.250	1	loss	9p22.1-p22.2
9	43,900,000	44,050,000	0.150	0	loss	9p11.2
9	73,300,000	73,350,000	0.050	1	loss	9q21.12
11	48,300,000	48,400,000	0.100	3	gain	11p11.2
11	50,350,000	50,750,000	0.400	3	gain	11p11.12

**Chromosome**	**Start**	**End**	**Length, Mb**	**Predicted copy number**	**Alteration**	**Cytoband**
12	28,750,000	28,800,000	0.050	3	gain	12p11.22
12	34,200,000	38,700,000	4.500	3	gain	12q11-q12
12	52,550,000	53,300,000	0.750	3	gain	12q13.13
12	57,900,000	58,100,000	0.200	3	gain	12q13.3
12	67,050,000	67,450,000	0.400	3	gain	12q14.3
12	67,450,000	68,950,000	1.500	3	gain	12q14.3-q15
12	68,950,000	70,700,000	1.750	3	gain	12q15
12	70,700,000	72,150,000	1.450	3	gain	12q15-q21.1
12	72,850,000	73,000,000	0.150	3	gain	12q21.1
13	26,600,000	63,600,000	37.000	1	loss	13q12.2-q21.1
13	63,650,000	115,100,000	51.450	1	loss	13q21.32-q21.33
14	19,150,000	19,600,000	0.450	1	loss	14q11.2
15	22,650,000	28,950,000	6.300	1	loss	15q13.1-q12
15	28,950,000	30,400,000	1.450	1	loss	15q13.2-q13.1
15	30,900,000	32,450,000	1.550	1	loss	15q13.3-q13.2
15	32,900,000	41,850,000	8.950	1	loss	15q13.3-q14
15	41,850,000	41,950,000	0.100	1	loss	15q15.1
15	42,550,000	42,600,000	0.050	1	loss	15q15.1
15	42,600,000	42,850,000	0.250	1	loss	15q15.1-q15.2
15	42,850,000	52,700,000	9.850	1	loss	15q21.2-q15.2

Chromosome	Start	End	Length, Mb	Predicted copy number	Alteration	Cytoband
15	52,700,000	67,050,000	14.350	1	loss	15q22.2-q22.1
16	5,950,000	14,750,000	8.800	3	gain	16p13.3-p13.12
16	16,850,000	17,650,000	0.800	3	gain	16p12.3
16	17,650,000	18,200,000	0.550	3	gain	16p12.3
16	18,750,000	21,400,000	2.650	3	gain	16p12.3-p12.2
16	22,600,000	28,350,000	5.750	3	gain	16p12.2-p12.1
16	33,900,000	34,000,000	0.100	9	gain	16p11.2
17	22,200,000	22,250,000	0.050	5	gain	17p11.2-p11.1
17	25,300,000	25,350,000	0.050	4	gain	17q11.1
18	15,150,000	15,400,000	0.250	3	gain	18p11.21
21	14,450,000	29,050,000	14.600	1	loss	21q11.2-q21.2
21	29,050,000	29,100,000	0.050	1	loss	21q21.3
21	29,100,000	48,100,000	19.000	1	loss	21q22.11-q21.3
22	22,600,000	22,700,000	0.100	3	gain	22q11.22
22	23,000,000	23,600,000	0.600	3	gain	22q11.22-q11.23
22	24,300,000	24,400,000	0.100	1	loss	22q11.23
22	24,400,000	24,550,000	0.150	3	gain	22q11.23
X	52,800,000	58,550,000	5.750	1	loss	Xp11.21-Xp11.22
X	108,250,000	108,300,000	0.050	3	gain	Xq22.3
Y	13,550,000	13,650,000	0.100	1	loss	Yq11.21

Supplementary Table S6 Chromosome-Y small-variant annotations

The table contains 71 chromosome-Y records with source coordinates, depths, and VAF values. Previous gene symbols were mapped to current HGNC symbols. Unique-mappability and male-specific-Y masking were not performed, and the reference assembly was not supplied. These selected-site annotations do not constitute a chromosome-wide dosage or coding-region assessment.

Chr	Position	REF	ALT	Class	Mutation class	Tumor VAF	Tumor depth	Normal VAF	Normal depth	Region	Gene annotation	Cytoband
chrY	3,074,136	T	C	SNV	Intergenic	0.500	19	0.000	16	intergenic	LINC00278;TGIF2LY	Yp11.2
chrY	3,596,100	G	T	SNV	Intergenic	0.700	13	0.000	11	intergenic	TGIF2LY;PCDH11Y	Yp11.2
chrY	4,062,973	C	A	SNV	Intergenic	0.667	8	0.000	14	intergenic	TGIF2LY;PCDH11Y	Yp11.2
chrY	4,075,497	A	G	SNV	Intergenic	0.556	12	0.000	18	intergenic	TGIF2LY;PCDH11Y	Yp11.2
chrY	4,386,778	T	G	SNV	Intergenic	0.417	13	0.000	15	intergenic	TGIF2LY;PCDH11Y	Yp11.2
chrY	4,537,046	C	A	SNV	Intergenic	1.000	3	0.000	15	intergenic	TGIF2LY;PCDH11Y	Yp11.2
chrY	4,985,711	A	T	SNV	Intronic	0.444	10	0.000	12	intronic	PCDH11Y	Yp11.2
chrY	4,985,900	T	C	SNV	Intronic	0.556	11	0.000	11	intronic	PCDH11Y	Yp11.2
chrY	5,409,177	A	G	SNV	Intronic	0.250	14	0.000	16	intronic	PCDH11Y	Yp11.2
chrY	5,412,537	G	T	SNV	Intronic	0.727	12	0.000	13	intronic	PCDH11Y	Yp11.2
chrY	5,460,729	C	A	SNV	Intronic	0.636	13	0.000	22	intronic	PCDH11Y	Yp11.2
chrY	5,496,671	A	T	SNV	Intronic	0.231	15	0.000	26	intronic	PCDH11Y	Yp11.2
chrY	5,751,706	T	C	SNV	Intergenic	0.600	22	0.000	20	intergenic	PCDH11Y;TTTY23	Yp11.2
chrY	6,474,229	A	G	SNV	Intergenic	0.625	10	0.000	21	intergenic	TTTY8;AMELY	Yp11.2
chrY	6,926,907	G	T	SNV	Intronic	0.250	14	0.000	17	intronic	TBL1Y	Yp11.2
chrY	6,940,200	C	A	SNV	Intronic	0.333	10	0.000	16	intronic	TBL1Y	Yp11.2
chrY	7,063,886	C	A	SNV	Intergenic	0.375	9	0.000	17	intergenic	TBL1Y;PRKY	Yp11.2
chrY	7,421,991	C	A	SNV	Intergenic	0.333	10	0.000	13	intergenic	PRKY;TTTY16	Yp11.2
chrY	7,818,830	G	C	SNV	Intergenic	0.385	15	0.000	16	intergenic	TTTY12;LINC00279	Yp11.2

Chr	Position	REF	ALT	Class	Mutation class	Tumor VAF	Tumor depth	Normal VAF	Normal depth	Region	Gene annotation	Cytoband
chrY	7,892,586	C	T	SNV	Intergenic	0.500	9	0.000	21	intergenic	TTTY12;LINC00279	Yp11.2
chrY	9,014,341	G	T	SNV	Intergenic	0.385	16	0.000	17	intergenic	TTTY11;RBMY1A3P	Yp11.2
chrY	9,112,624	GGAGGAA	G	INDEL	Intergenic	0.778	9	0.000	21	intergenic	TTTY11;RBMY1A3P	Yp11.2
chrY	9,377,617	G	T	SNV	Intergenic	0.273	11	0.000	11	intergenic	TSPY4;RBMY3AP	Yp11.2
chrY	9,446,871	T	A	SNV	Intergenic	0.846	15	0.000	22	intergenic	TSPY4;RBMY3AP	Yp11.2
chrY	9,908,669	A	G	SNV	Intergenic	0.429	7	0.000	18	intergenic	TTTY23;NONE	Yp11.2
chrY	10,013,019	G	C	SNV	Intergenic	0.179	46	0.000	38	intergenic	TTTY23;NONE	Yp11.2
chrY	13,266,638	G	A	SNV	Intergenic	0.087	47	0.000	90	intergenic	NONE;GYG2P1	Yq11.1
chrY	13,278,401	A	T	SNV	Intergenic	0.321	28	0.000	52	intergenic	NONE;GYG2P1	Yq11.1
chrY	13,293,688	A	G	SNV	Intergenic	0.069	88	0.000	99	intergenic	NONE;GYG2P1	Yq11.1
chrY	13,545,981	A	C	SNV	Intergenic	0.333	9	0.000	9	intergenic	NONE;GYG2P1	Yq11.21
chrY	13,545,982	T	C	SNV	Intergenic	0.333	9	0.000	10	intergenic	NONE;GYG2P1	Yq11.21
chrY	13,634,919	A	T	SNV	Intergenic	0.194	40	0.025	50	intergenic	NONE;GYG2P1	Yq11.21
chrY	13,657,767	G	T	SNV	Intergenic	0.250	14	0.000	17	intergenic	NONE;GYG2P1	Yq11.21
chrY	13,905,826	G	T	SNV	Intergenic	0.333	10	0.000	18	intergenic	NONE;GYG2P1	Yq11.21
chrY	13,913,951	AC	A	INDEL	Intergenic	1.000	4	0.000	19	intergenic	NONE;GYG2P1	Yq11.21
chrY	14,271,648	C	A	SNV	Intergenic	0.300	12	0.000	20	intergenic	NONE;GYG2P1	Yq11.21
chrY	15,279,170	A	T	SNV	Intergenic	1.000	6	0.000	10	intergenic	DDX3Y;UTY	Yq11.221
chrY	15,352,488	C	T	SNV	Intergenic	0.444	10	0.000	18	intergenic	DDX3Y;UTY	Yq11.221
chrY	15,398,477	T	C	SNV	Intronic	0.538	13	0.000	14	intronic	UTY	Yq11.221
chrY	15,701,842	C	CA	INDEL	Intergenic	0.364	15	0.000	14	intergenic	UTY;TMSB4Y	Yq11.221

Chr	Position	REF	ALT	Class	Mutation class	Tumor VAF	Tumor depth	Normal VAF	Normal depth	Region	Gene annotation	Cytoband
chrY	15,835,602	A	G	SNV	Intergenic	0.286	20	0.000	20	intergenic	TMSB4Y;VCY1B	Yq11.221
chrY	15,901,933	G	A	SNV	Intergenic	0.900	13	0.000	13	intergenic	TMSB4Y;VCY1B	Yq11.221
chrY	15,911,304	G	T	SNV	Intergenic	0.500	7	0.000	23	intergenic	TMSB4Y;VCY1B	Yq11.221
chrY	16,001,758	C	A	SNV	Intergenic	0.300	10	0.000	23	intergenic	TMSB4Y;VCY1B	Yq11.221
chrY	16,367,642	T	G	SNV	Intergenic	1.000	16	0.000	9	intergenic	VCY;NLGN4Y	Yq11.221
chrY	16,377,554	C	A	SNV	Intergenic	0.444	22	0.000	21	intergenic	VCY;NLGN4Y	Yq11.221
chrY	17,185,367	T	A	SNV	Intergenic	0.769	18	0.000	16	intergenic	NLGN4Y;NONE	Yq11.221
chrY	17,490,332	G	A	SNV	Intergenic	0.615	13	0.000	13	intergenic	NLGN4Y;NONE	Yq11.221
chrY	17,632,318	C	A	SNV	Intergenic	0.375	11	0.000	23	intergenic	NLGN4Y;FAM41AY1	Yq11.221
chrY	17,749,522	C	A	SNV	Intergenic	0.500	10	0.000	17	intergenic	NLGN4Y;FAM41AY1	Yq11.221
chrY	17,873,263	CA	C	INDEL	Intergenic	0.875	12	0.000	11	intergenic	NLGN4Y;FAM41AY1	Yq11.221
chrY	17,953,855	T	C	SNV	Intergenic	1.000	14	0.043	29	intergenic	NLGN4Y;FAM41AY1	Yq11.221
chrY	21,789,054	T	A	SNV	Intergenic	0.750	17	0.000	12	intergenic	TXLNGY;KDM5D	Yq11.222
chrY	21,926,094	C	A	SNV	Intergenic	0.300	11	0.000	15	intergenic	KDM5D;TTTY10	Yq11.222
chrY	22,186,936	G	T	SNV	Intergenic	0.333	9	0.000	21	intergenic	KDM5D;TTTY10	Yq11.223
chrY	22,237,062	A	C	SNV	Intergenic	0.550	27	0.000	30	intergenic	KDM5D;TTTY10	Yq11.223
chrY	22,439,591	C	A	SNV	Intergenic	0.179	38	0.000	46	intergenic	KDM5D;TTTY10	Yq11.223
chrY	23,083,937	G	T	SNV	Intergenic	0.667	13	0.000	12	intergenic	RPS4Y2;PRORY	Yq11.223
chrY	23,733,204	TA	T	INDEL	Intergenic	0.733	19	0.000	19	intergenic	RBMY1D;TTTY13	Yq11.223
chrY	24,727,953	G	A	SNV	Intergenic	0.600	7	0.000	14	intergenic	PRY2;TTTY17B	Yq11.223
chrY	25,604,551	G	A	SNV	Intergenic	0.429	34	0.000	36	intergenic	DAZ4;TTTY3B	Yq11.223
chrY	26,418,872	G	T	SNV	Intergenic	0.625	10	0.000	29	intergenic	GOLGA2P3Y;TTTY17A	Yq11.23

Chr	Position	REF	ALT	Class	Mutation class	Tumor VAF	Tumor depth	Normal VAF	Normal depth	Region	Gene annotation	Cytoband
chrY	28,568,976	G	T	SNV	Intergenic	0.300	16	0.000	22	intergenic	TTTY3B;NONE	Yq11.23
chrY	28,617,035	G	T	SNV	Intergenic	0.333	9	0.000	12	intergenic	TTTY3B;NONE	Yq11.23
chrY	28,688,445	T	A	SNV	Intergenic	0.500	18	0.000	28	intergenic	TTTY3B;NONE	Yq11.23
chrY	58,968,359	C	A	SNV	Intergenic	0.048	128	0.000	129	intergenic	NONE;SPRY3	Yq12
chrY	58,983,596	T	C	SNV	Intergenic	0.103	151	0.000	191	intergenic	NONE;SPRY3	Yq12
chrY	58,986,901	T	C	SNV	Intergenic	0.167	65	0.011	103	intergenic	NONE;SPRY3	Yq12
chrY	59,008,980	T	C	SNV	Intergenic	0.095	77	0.011	108	intergenic	NONE;SPRY3	Yq12
chrY	59,012,354	T	A	SNV	Intergenic	0.167	161	0.000	239	intergenic	NONE;SPRY3	Yq12
chrY	59,025,300	A	G	SNV	Intergenic	0.039	177	0.005	236	intergenic	NONE;SPRY3	Yq12

Supplementary Table S7 Functional-category reconciliation

The eight categories in the original Figure 4B refer to selected categories from the whole small-variant summary, not to eight components of the 2,714-record subset. The table below distinguishes arithmetic checks from a validated consequence-level reconstruction.

Reported category or comparison	Count	Provenance or limitation
Intergenic	16002	Original Figure 4B
Intronic	10140	Original Figure 4B
ncRNA	1933	Original Figure 4B
Splice-associated	1418	Original Figure 4B
Frameshift deletion	605	Original Figure 4B
UTR	576	Original Figure 4B
Upstream/downstream	339	Original Figure 4B
Missense	213	Original Figure 4B
Sum of eight displayed categories	31226	Calculated from displayed counts
Whole reported small-variant set	31877	Original Figure 4A / Table 3
Not represented by eight displayed counts	651	Arithmetic difference; classes unavailable
Previously reported selected subset	2714	Membership/consequence criteria unresolved
Splice + frameshift deletion + missense	2236	Potentially relevant classes; subset membership unverified
Difference from reported subset	478	Arithmetic only; not a reconstructed functional class
Previously reported gene-expanded count	2739	Includes multiple assignments; not a distinct-variant count

The source documentation did not include the annotation tool, version, exact transcript/consequence fields, or essential-splice definition.

Supplementary Table S8 Sequencing information and analytical availability

Component	Required information	Availability
Sample design	One tumor–blood pair from A14, age 60	Reported; molecular identity fingerprint unavailable
Platform and library preparation	Platform, read length, kit, DNA preparation, and duplicate handling	Unavailable
Reference	Assembly, patch level, reference sequence, coordinate conventions	Unavailable
Alignment	Aligner, version, parameters, alignment files	Unavailable
Small-variant calling	Caller, version, PASS criteria, complete allele-level VCF	Unavailable
Annotation	Tool, version, transcript set, consequence-selection rules	Unavailable

Component	Required information	Availability
Coverage	Separate tumor/normal genome-wide mean/median and ≥20×/≥30× fractions	Unavailable; original 38× summary is not genome-wide QC
Technical QC	Duplicate rate, insert size, cross-sample contamination, genotype concordance	Unavailable
Orientation artifacts	F1R2/F2R1 and OXOG metrics; orientation filtering	Not performed; reads unavailable
Signatures and burden	Trinucleotide decomposition and callable-genome TMB	Not estimable from available summaries
Purity and copy number	Purity/ploidy, BAF/log-R, sex-chromosome baseline	Unavailable; integer segment labels retained
SV filtering	Complete length distribution, normal panel, gnomAD-SV/DGV and mappability filtering	Not performed; complete calls unavailable
Population frequencies	Variant-level gnomAD/dbSNP matching with verified assembly	Not performed
CTNNB1 and APC	Callable exon-level assessment and germline classification	Undetermined
Orthogonal validation	Breakpoint PCR/Sanger, RNA, SNP array, FISH or other copy-number confirmation	Not performed
Deposition	Filtered VCF/raw-read accession	Not deposited; no accession available

Supplementary Table S9 Comparison with the published exome study

Feature	Kumari et al. 2024	Present annotation review
Design	13 NA patients and 5 male controls	One reported tumor–blood pair
Clinical setting	Advanced-stage JNA; mean age 13.8 ± 2.4 years	A14; 60 years; stage IIIB
Y coding findings	28 positions in 14 genes in 9 patients	No validated callable coding result available
Reported noncoding Y count	Not used as the comparison endpoint	71 source annotations
USP9Y, UTY, KDM5D, DDX3Y, TSPY4	Highlighted in the published abstract	Locus-specific coverage/status not established
Burden or pathway comparison	WES-based study-specific targets and methods	No matched callable denominator or validated somatic set
Interpretation	Published coding-region observations	Cannot establish replication, negative findings, or age-related differences

Source: Kumari et al. Genes Chromosomes Cancer 2024;63:e23265. DOI: 10.1002/gcc.23265; PMID: 39297564. The numerical comparison is limited to the verified published abstract; unverified whole-exome class totals are not supplied.

Supplementary Table S10 Gene-symbol audit

All 278 distinct identifiers in the available gene, breakend, and chromosome-Y columns were checked against the HGNC complete set retrieved on 15 September 2026. Thirteen previous symbols had unambiguous approved replacements; nine LOC identifiers had no approved-symbol match. No date-like conversion was detected in

these extracted text fields. The unavailable upstream workbook was not screened. All unchanged approved identifiers and the audit source are documented in the accompanying CSV.

**Original symbol**	**Current symbol**	**HGNC identifier**
ATP5B	ATP5F1B	HGNC:830
C11orf80	TOP6BL	HGNC:26197
C1orf127	CIROZ	HGNC:26730
FAM69A	DIPK1A	HGNC:32213
H3F3B	H3-3B	HGNC:4765
H3F3C	H3-5	HGNC:33164
IARS	IARS1	HGNC:5330
ILVBL	HACL2	HGNC:6041
MARCH1	MARCHF1	HGNC:26077
MFSD10	SLC75A1	HGNC:16894
NCL	NUCLEOLIN	HGNC:7667
SEPT11	SEPTIN11	HGNC:25589
TMEM5-AS1	RXYLT1-AS1	HGNC:48910

Identifiers without an approved-symbol match: LOC100128164, LOC100130331, LOC100507291, LOC101928372, LOC101929095, LOC105371703, LOC105377967, LOC341056, LOC407835.

HGNC source: https://www.genenames.org/ and https://storage.googleapis.com/public-download-files/hgnc/tsv/tsv/hgnc_complete_set.txt. Previous symbols are retained in the audit to preserve traceability.
